# Surgical treatment for far-out syndrome associated with abnormal fusion of the L5 vertebral corpus and L4 hemivertebra: a case report

**DOI:** 10.1186/s13104-016-2123-2

**Published:** 2016-06-28

**Authors:** Shohei Ise, Koki Abe, Sumihisa Orita, Tetsuhiro Ishikawa, Kazuhide Inage, Kazuyo Yamauchi, Miyako Suzuki, Jun Sato, Kazuki Fujimoto, Yasuhiro Shiga, Hirohito Kanamoto, Masahiro Inoue, Hideyuki Kinoshita, Kazuhisa Takahashi, Seiji Ohtori

**Affiliations:** Department of Orthopaedic Surgery, Graduate School of Medicine, Chiba University, 1-8-1 Inohana, Chuo-ku, Chiba, Chiba 260-8670 Japan; Department of Orthopaedic Surgery, Sanmu Medical Center, 167 Naruto, Sanmu, Chiba 289-1326 Japan

**Keywords:** Far-out syndrome, Assimiration vertebra, Congenital deformity, Lumbar extraforaminal entrapment, L5 nerve root

## Abstract

**Background:**

Far-out syndrome was reported by Wiltse et al. in 1984, which is a condition characterized by L5 spinal nerve radiculopathy due to nerve compression between the L5 transverse process and sacral alar. Although many cases of far-out syndrome have been reported, to our knowledge, the present case firstly showed far-out syndrome due to assimilated L4 hemivertebra and L5 vertebra through which abnormal nerve root passed.

**Case presentation:**

A 71-year-old man presented with left lower back pain and intermittent claudication accompanied by severe left buttock pain. Radiological examination showed assimilation between the L4 hemivertebra and L5 vertebra, which had two pedicles on the right side, with no canal stenosis. However, computed tomography and magnetic resonance imaging of coronal sections showed extraforaminal stenosis between the L5 transverse process and sacral alar, whereby the L5 spinal nerve was pinched (“far-out lesion”), and an abnormal nerve root passage in the assimilated vertebral corpus. We performed transforaminal lumbar interbody fusion, then resected the L5 transverse process to decompress the extraforaminal stenosis, and finally installed pedicle screws, but not at the one of pedicles of the assimilated vertebra in order to prevent nerve injury. Postoperatively, the patient had no symptoms up to 1.5 years after the surgery.

**Conclusion:**

The current case suggests the importance of detailed preoperative examination of patients with anatomical abnormalities such as assimilated vertebrae, which may result in incorrect diagnosis and failed surgery.

## Background

Far-out syndrome, first reported by Wiltse et al. in 1984 [1], is a condition characterized by L5 spinal nerve radiculopathy due to nerve compression between the L5 transverse process and sacral alar. Extraforaminal lesions of far-out syndrome are known to be difficult to diagnose, and spinal deformities such as spondylosis, disc degeneration and congenital abnormalities tend to be comorbidities [1–4]. Here, we present the case of a patient who showed a rare anatomical feature, that is, a unique vertebral assimilation, which caused far-out syndrome.

## Case presentation

**Fig. 1:**
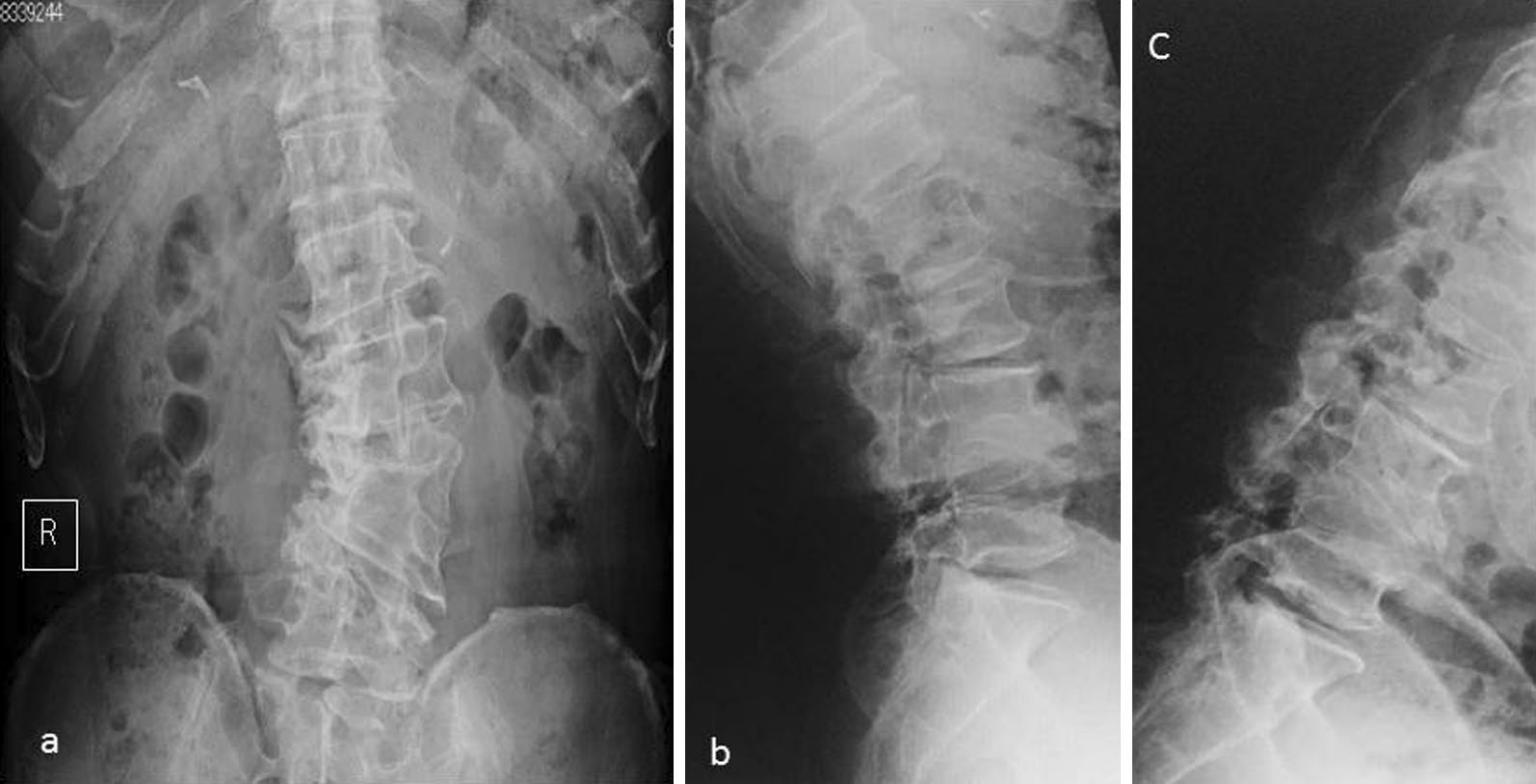
**a** *Anteroposterior-view* plain X-ray scan of the lumbar spine showing scoliosis accompanied by degenerative changes and two assimilation vertebrae, one of which comprised the right L4 hemivertebra and L5 vertebra and the other comprised the left L4 hemivertebra and L3 vertebra. **b**, **c** *Lateral-view* plain X-ray scan of the lumbar spine in flexion and extension positions showing L5–S1 instability with 10° sagittal rotation

**Fig. 2:**
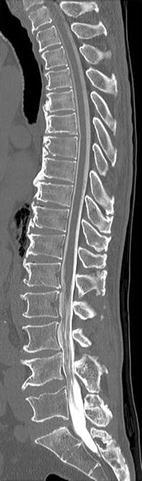
Computed tomographic scan of sagittal section followed by myelography showing no stenosis in the central spinal canal

**Fig. 3:**
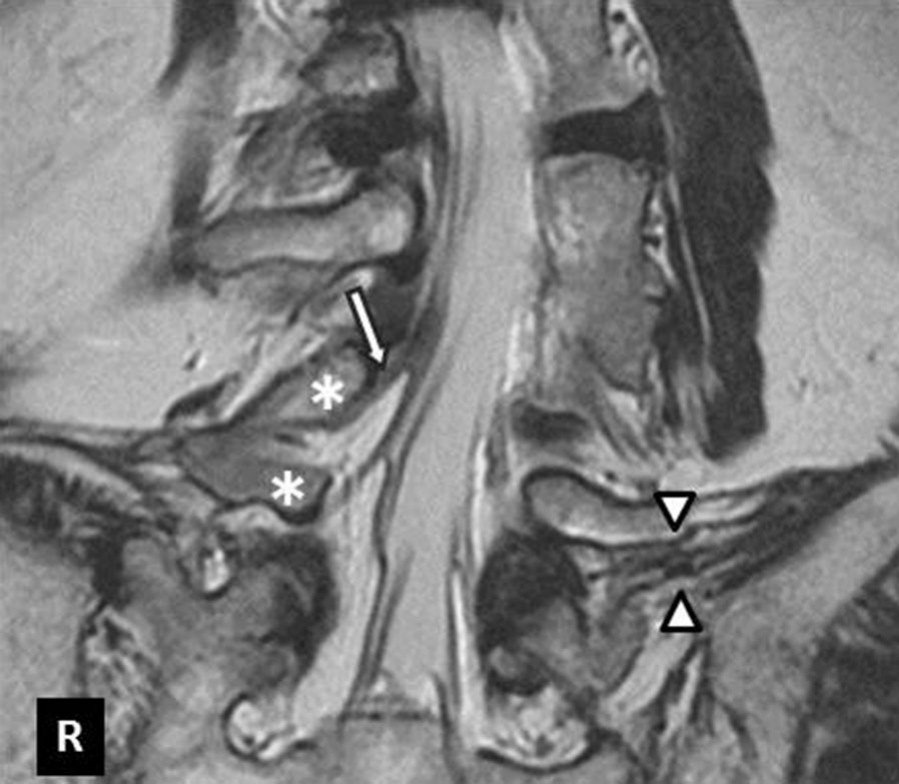
T2-weighted magnetic resonance image of coronal section. The *two* *white triangles* indicate the left L5 root compressed between the transverse process and sacral alar. The *asterisks* indicate the two left pedicles of the fused L5 vertebra. The *white arrow* indicates the right L4 nerve root

**Fig. 4:**
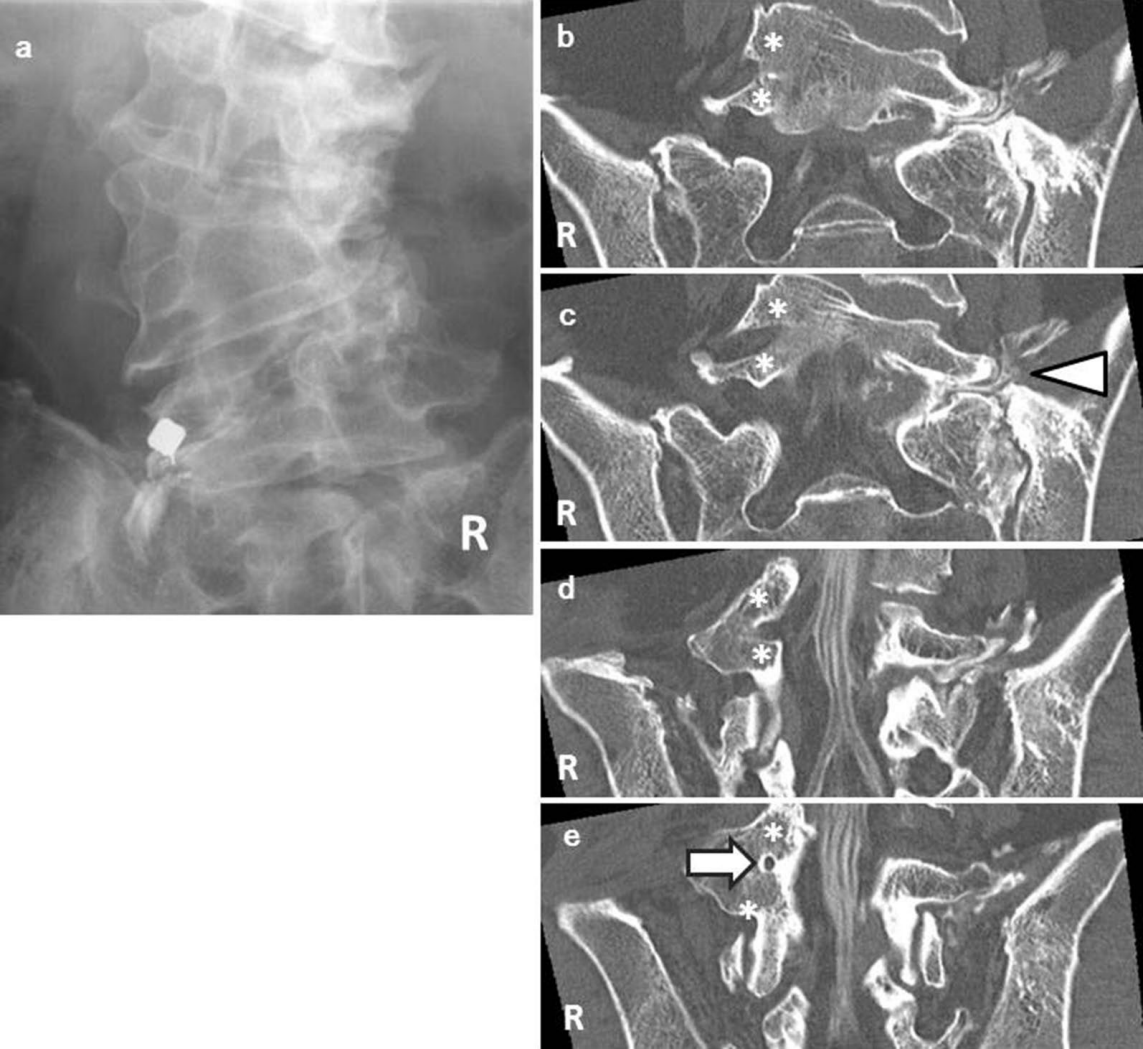
**a** Selective radiculography of the left L5 spinal nerve. **b**–**e** Computed tomographic scans of the coronal section. The *asterisks* indicate the two left pedicles of the fused L5 vertebra. **c** The *white arrowheads* show the left L5 spinal nerve pinched between the transverse process and sacral alar. **e** The *white arrow* indicates a unique foremen “like a bony tunnel” through which the left L5 spinal nerve emerges

**Fig. 5:**
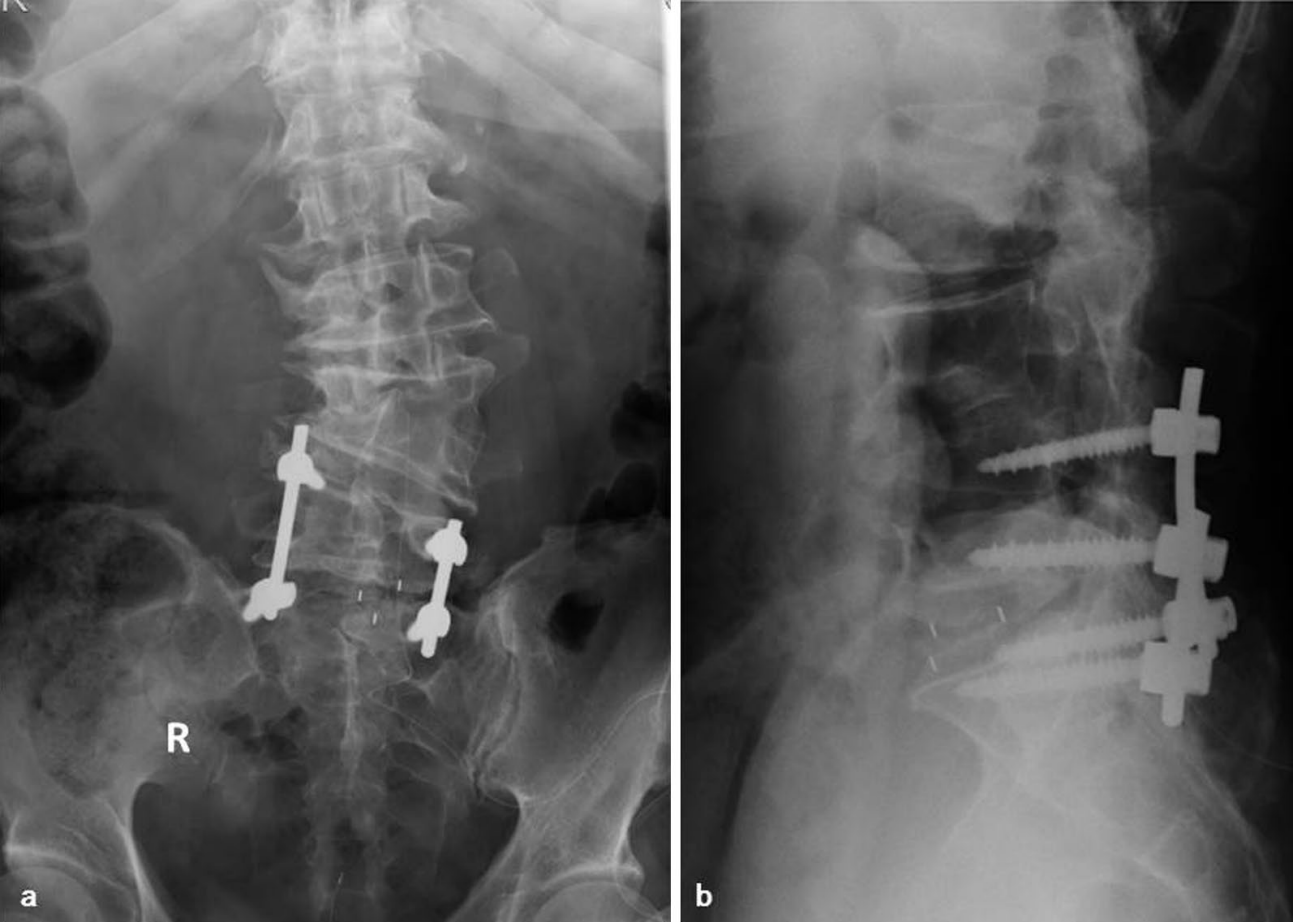
**a**, **b** Postoperative plain X-ray scans, anteroposterior and lateral views, respectively

A 71-year-old man presented with an over 10-year history of left lower back pain and intermittent claudication accompanied by severe pain radiating to the left buttock. Physical examination confirmed hyperesthesia in the left hip but no apparent motor weakness. On both sides, the straight leg raising test yielded negative results. A plain X-ray scan showed L4–5 assimilated vertebra and junctional scoliosis. Flexion and extension views showed lumbosacral instability with 10° sagittal rotation (Fig. 1). Magnetic resonance imaging (MRI) and myelography followed by computed tomographic scan (CT) showed no central spinal stenosis (Fig. 2). However, MRI of the coronal section indicated stenosis of the left L5 spinal nerve far outside the L5–S1 foramen (Fig. 3). Further, CT following radiculography for the left L5 spinal nerve showed apparent pinching of the left L5 spinal nerve between the L5 transverse process and sacral alar (Fig. 4). Selective L5 spinal nerve infiltration relieved pain for only a day. We diagnosed the patient with far-out syndrome based on the abovementioned evidence, specifically, the pinching of the left L5 spinal nerve between the L5 transverse process and sacral alar. We performed transforaminal lumbar interbody fusion (TLIF) surgery to decompress the left L5 spinal nerve and stabilize the lumbosacral junction following Wiltse’s approach [1]. Intraoperative left L5–S1 facetectomy revealed severe compression of the L5 spinal nerve between the transverse process and sacral alar, as expected from the preoperative observations. We decompressed the nerve by cutting off the inferior margin of the transverse process and superior margin of the sacral alar. We then inserted an intervertebral cage filled with autologous bone into the L5–S1 intervertebral space for lumbosacral stabilization and to enlarge the lumbosacral foramen, followed by pedicle screw fixation, which required particular care because of the abnormal anatomy. Preoperative CT and MRI had already shown that the fused L5 vertebral body had two pedicles on its right side: the cranial one originated from the L4 vertebra and the caudal one was an anatomically appropriate L5 pedicle. The right L4 root passed between the two right pedicles of the fused L5 vertebral body, as if it ran through “a bony tunnel” (Figs. 3, 4). Moreover even the C-arm imaging system couldn’t precisely show the right root passage in the fused vertebra. Therefore, we took great care while inserting the pedicle screw into the cranial pedicle on the right of the L5 so as not to injure the right L4 root. Although the caudal pedicle on the right of the L5 vertebra was left as is with no screw, the L5 and sacral vertebrae were fixed firmly (Fig. 5). Postoperatively, the patient had no symptoms and had a good course up to 1.5 years after the surgery.

## Discussion

Since Wiltse’s first report of far-out syndrome, many cases with similar pathological condition have been reported [1–9]. However, we could not detect one due to assimilation between L4 hemibertebra and L5 vertebra through which abnormal nerve passes. According to these reports, the syndrome was diagnosed by X-ray examination, CT, MRI, myelography, selective radiculography, and spinal nerve infiltration. Recently, electrography and diffusion tensor imaging have also been reported to be effective for diagnosing stenosis outside of the intervertebral foramen [2, 3, 10]. Most of the reported cases were treated by decompression and stabilization surgery. Nowadays, minimally invasive surgery (MIS), including mini-open TLIF and endoscopic extraforaminotomy, is preferred for this condition [7].

With regard to congenital vertebral deformities in the lumbosacral junction, previous reports have described some cases of pathology-associated lower back and leg pain. For example, Yoshioka et al. reported the case of a 39-year-old man with congenital absence of the L5–S1 facet joint and a conjoined nerve root who was treated by decompression and fixation surgery. This pathology was not detectable on plain X-ray scans but was observed in CT and MRI [11]. Similarly, a 31-year-old man who complained of lower back pain and disturbed gait because of a dorsal midline hemivertebra was evaluated by sagittal reconstructed CT and successfully underwent an elaborate surgery for treatment [12].

Generally, established surgical procedures such as MIS can be used to treat pathological lesions in normal anatomical structures, because the degenerative changes are predictable to some extent. However, in the case of congenital anatomical deformities, detailed preoperative imaging tests and well-planned elaborate surgeries are needed on a case-by-case basis.

The current case was a unique one of far-out syndrome with anatomical abnormality due to assimilated vertebrae. At the first visit, the patient was suspected to have simple canal stenosis because of the intermittent claudication and the observation of assimilated vertebrae and regional scoliosis on plain X-ray scans. However, CT and MRI of coronal sections and myelography following CT showed no canal stenosis and instead indicated stenosis outside of the L5–S1 intervertebral foramen, that is, a “far-out lesion.” Finally, enhancement and infiltration of the L5 spinal nerve confirmed the final diagnosis. To treat the severe stenosis at the far-out lesion with the assimilated vertebrae, we decided to perform rigid intervertebral fusion with adequate bone resection, which completely relieved the patient’s pain. In addition, detailed preoperative CT and MRI investigations facilitated successfully screw insertion into one of the right L5 pedicles, which was located in an anatomically complex lesion.

The current case suggests the importance of detailed preoperative examination of patients with anatomical abnormalities such as assimilated vertebrae, which sometimes result in incorrect diagnosis and failed surgery.

## Conclusions

We described the case of a patient with far-out syndrome associated with a uniquely deformed L5 vertebra involving an abnormal nerve root passage. CT and MRI examination of coronal sections were useful to diagnose this anatomical condition. After detailed preoperative imaging, we successfully performed TLIF surgery.

## Abbreviations

MRI: magnetic resonance imaging
CT: computed tomographic scan
TLIF: transforaminal lumbar interbody fusion
MIS: minimally invasive surgery
